# Synthesis, characterization, and bioactivity of carboxylic acid-functionalized titanium dioxide nanobelts

**DOI:** 10.1186/s12989-014-0043-7

**Published:** 2014-09-02

**Authors:** Raymond F Hamilton, Nianqiang Wu, Chengcheng Xiang, Ming Li, Feng Yang, Michael Wolfarth, Dale W Porter, Andrij Holian

**Affiliations:** 1 Center for Environmental Health Sciences, Department of Biomedical and Pharmaceutical Sciences, University of Montana, Missoula, 59812, MT, USA; 2 Department of Mechanical and Aerospace Engineering, West Virginia University, Morgantown, 26506-6106, WV, USA; 3 Department of Industrial and Management Systems Engineering, West Virginia University, Morgantown, 26506-6107, WV, USA; 4 National Institute for Occupational Safety and Health, Morgantown, 26506-6106, WV, USA

## Abstract

**Background:**

Surface modification strategies to reduce engineered nanomaterial (ENM) bioactivity have been used successfully in carbon nanotubes. This study examined the toxicity and inflammatory potential for two surface modifications (humic acid and carboxylation) on titanium nanobelts (TNB).

**Methods:**

The *in vitro* exposure models include C57BL/6 alveolar macrophages (AM) and transformed human THP-1 cells exposed to TNB for 24 hrs in culture. Cell death and NLRP3 inflammasome activation (IL-1β release) were monitored. Short term (4 and 24 hr) *in vivo* studies in C57BL/6, BALB/c and IL-1R null mice evaluated inflammation and cytokine release, and cytokine release from *ex vivo* cultured AM.

**Results:**

Both *in vitro* cell models suggest that the humic acid modification does not significantly affect TNB bioactivity, while carboxylation reduced both toxicity and NLRP3 inflammasome activation. In addition, short term *in vivo* exposures in both C57BL/6 and IL-1R null mouse strains demonstrated decreased markers of inflammation, supporting the *in vitro* finding that carboxylation is effective in reducing bioactivity. TNB instillations in IL-1R null mice demonstrated the critical role of IL-1β in initiation of TNB-induced lung inflammation. Neutrophils were completely absent in the lungs of IL-1R null mice instilled with TNB for 24 hrs. However, the cytokine content of the IL-1R null mice lung lavage samples indicated that other inflammatory agents, IL-6 and TNF-α were constitutively elevated indicating a potential compensatory inflammatory mechanism in the absence of IL-1 receptors.

**Conclusions:**

Taken together, the data suggests that carboxylation, but not humic acid modification of TNB reduces, but does not totally eliminate bioactivity of TNB, which is consistent with previous studies of other long aspect ratio nanomaterials such as carbon nanotubes.

## Background

The physical-chemical properties of engineered nanomaterials (ENM) are strongly dependent on size [1]–[3], shape [4]–[6] and surface chemistry [7]. Therefore, the size, shape and surface chemistry of ENM have been tailored to meet the practical need. For example, single-crystalline titanium dioxide nanobelts (TNB) have better photocatalytic activity than round-shaped titanium dioxide nanospheres (TNS) [8], since TNB have a lower charge recombination rate and better affinity with oxygen molecules as compared to TNS. Hence, TNB have great advantages in applications in catalysis, environmental remediation and sunscreen windows. In addition, TNB have better charge transport properties than TNS [9], which have promising applications in solar cells. Furthermore, titanium nanoparticles functionalized with different organic monolayers exhibit different behaviors in aggregation and surface adsorption in aqueous environments [7]. In particular, COOH-functionalized (COOH) titanium nanoparticles are more hydrophilic than bare particles. The variation of physical-chemical properties consequently leads to changes in bioactivity and toxicity of ENM.

The bioactivity of titanium nanoparticles is also correlated with both size and shape, with the longer TNB showing more bioactivity in both *in vivo* and *in vitro* exposure models [10],[11]. The proposed mechanism of TNB action is consistent with other bioactive ENM, first proposed for uric acid crystals, crystalline SiO_2_ and asbestos [12]. This cellular mechanism involves, in sequential order, particle uptake by macro pinocytosis, phago-lysosomal disruption, release of cathepsin B, and activation of the NLRP3 inflammasome assembly [13]. This, in turn, results in the sustained release of inflammatory cytokines IL-1β and IL-18 [14]. The longer, rigid ENM are resistant to normal lung clearance mechanisms, and a cycle of inflammation is established similar to that seen in MWCNT-exposures [15]–[18]. The role of autophagy in TNB-initiated lung inflammation is not understood yet, but like other bioactive ENM [13], the induction of autophagy is highly likely due to intracellular damage caused by the TNB [11].

One way to modify the bioactivity of TNB is to change the surface chemistry. The most frequently used method of ENM surface modification involves surface modification with carboxyl (-COOH) groups [19],[20]. This modification has been shown to significantly reduce ENM bioactivity in MWCNT exposures [21]–[23]. The purpose of this study was to investigate the possibility that side-wall functionalization of TNB could attenuate bioactivity and subsequent NLRP3 inflammasome activation and IL-1β release. Two TNB surface modifications, the covalent attachment of carboxyl groups (TNB-COOH) and the humic acid groups (TNB-HA), were tested in a variety of *in vitro* and *in vivo* mouse exposure models, in addition to a human macrophage cell line (transformed THP-1).

## Results

### Particle synthesis and characterization

**Figure 1:**
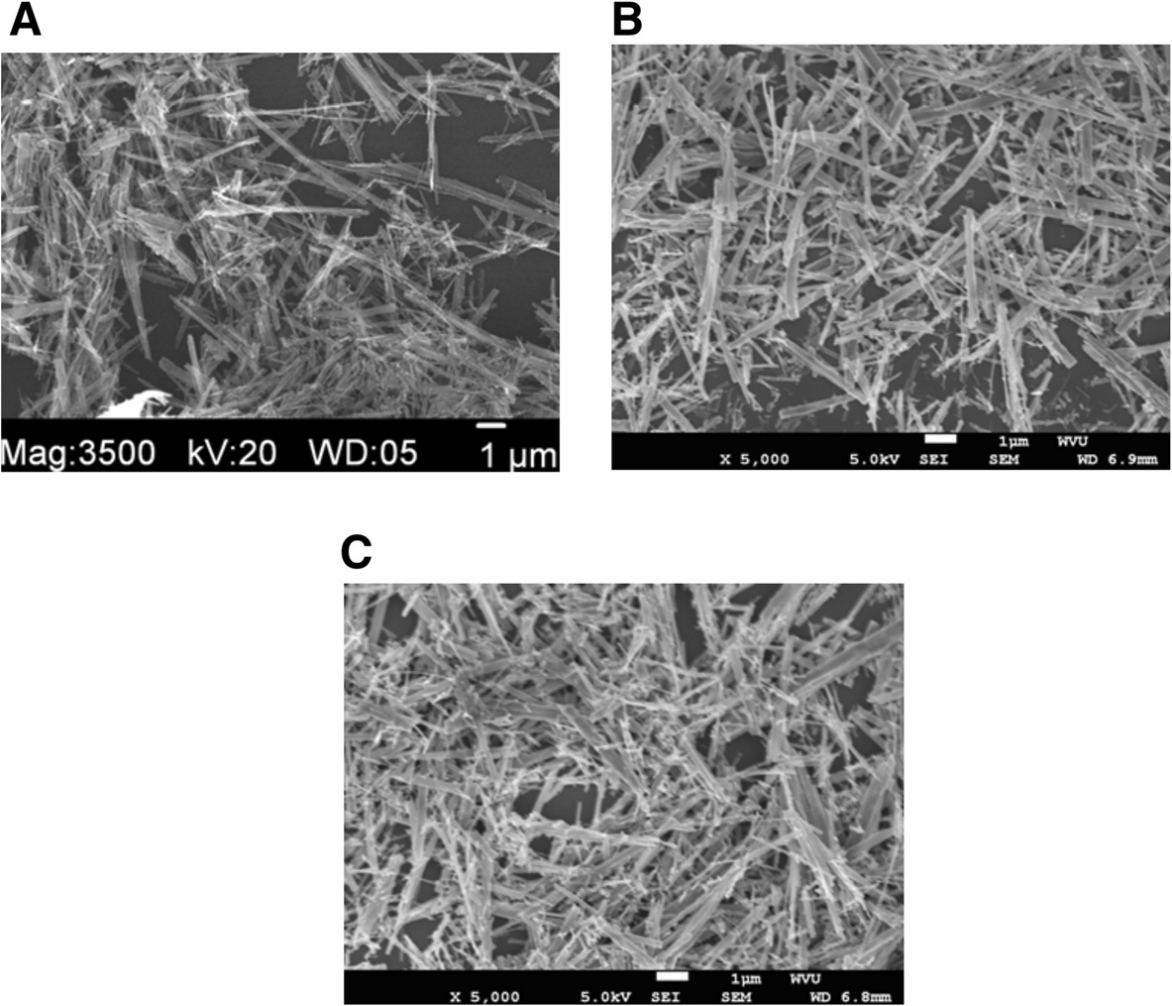
**SEM image of TiO**_**2**_**nanomaeterials; (A) the bare nanobelts, (B) the COOH-functionalized nanobelts, (C) the humic acid-coated nanobelts.**

**Figure 2:**
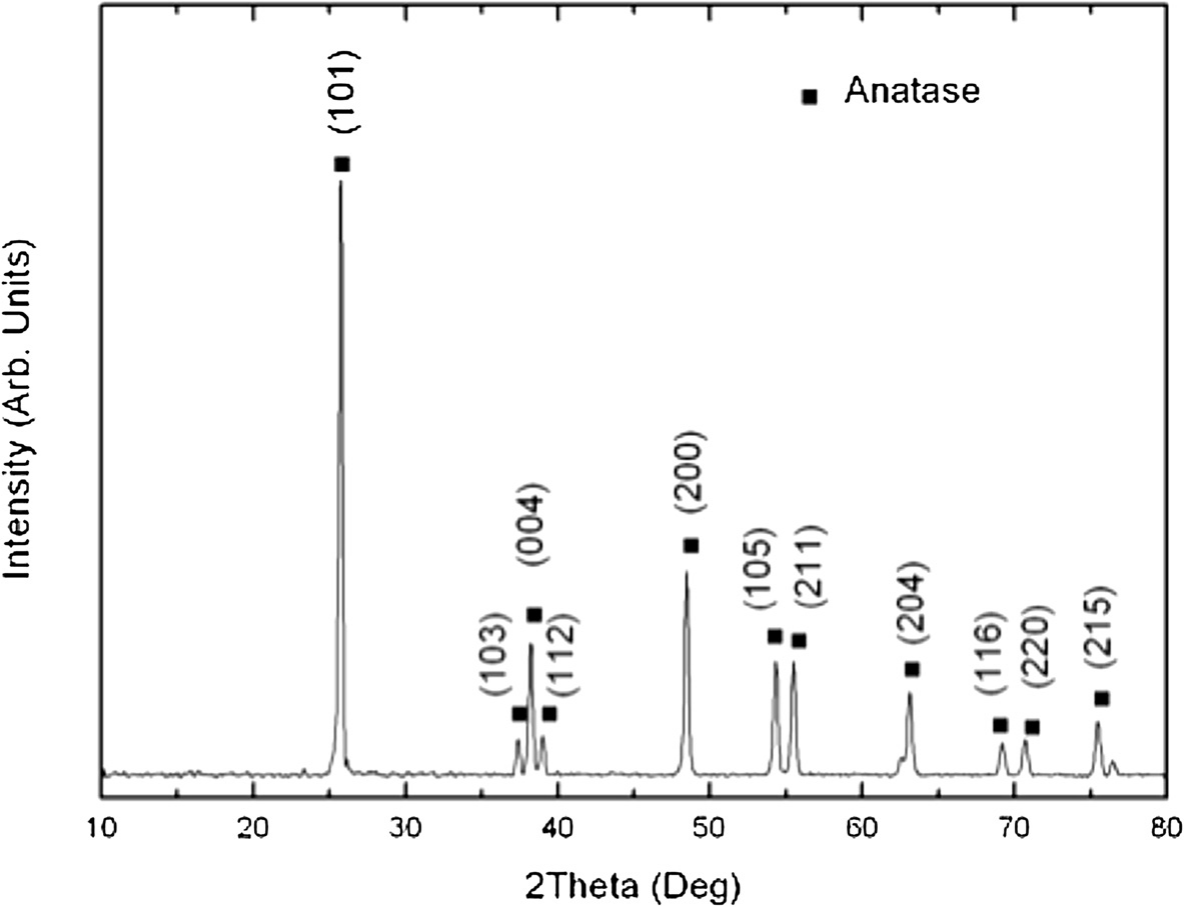
**XRD pattern of the bare TiO**_**2**_**nanobelts.**

**Figure 3:**
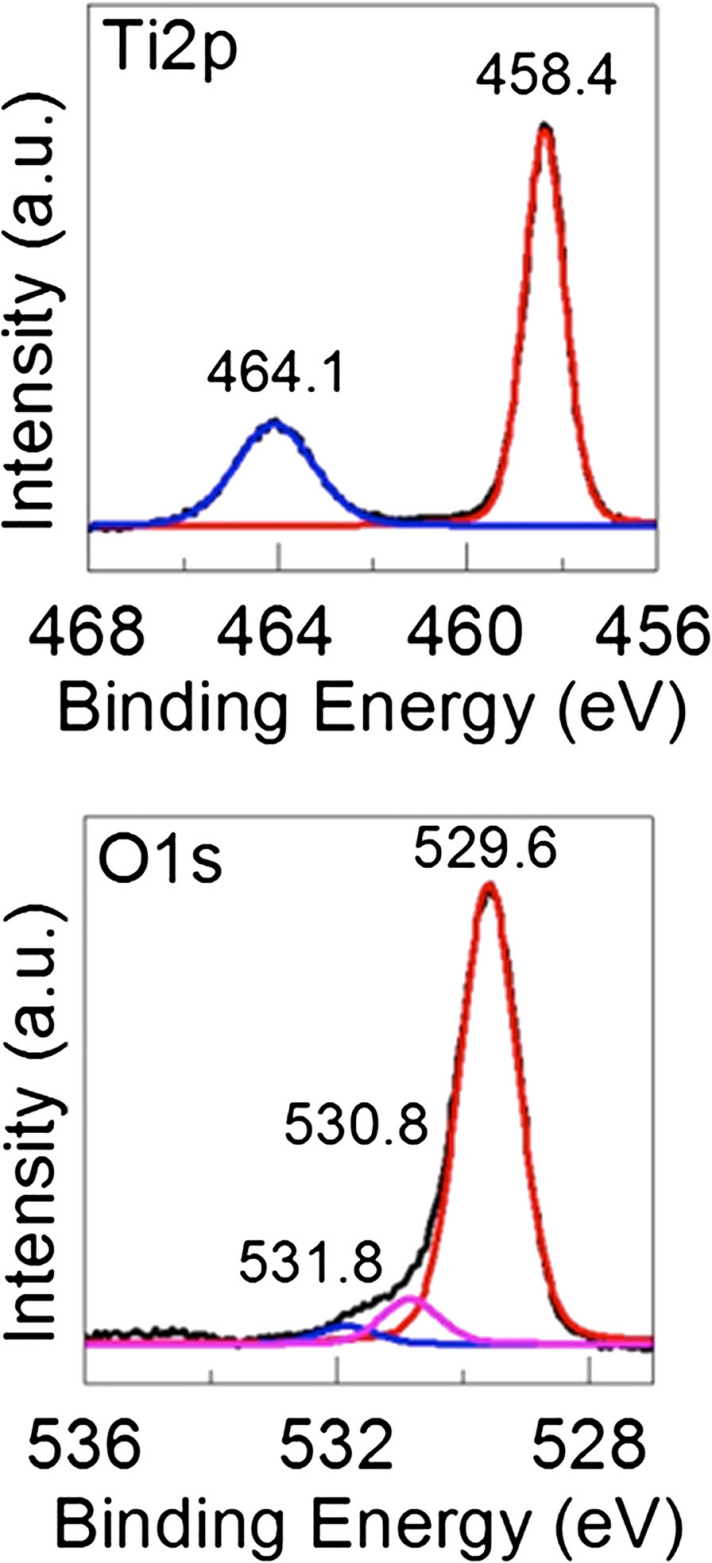
**Ti 2p and O 1 s core levels of the XPS spectra obtained from the bare TiO**_**2**_**nanobelts.**

**Figure 4:**
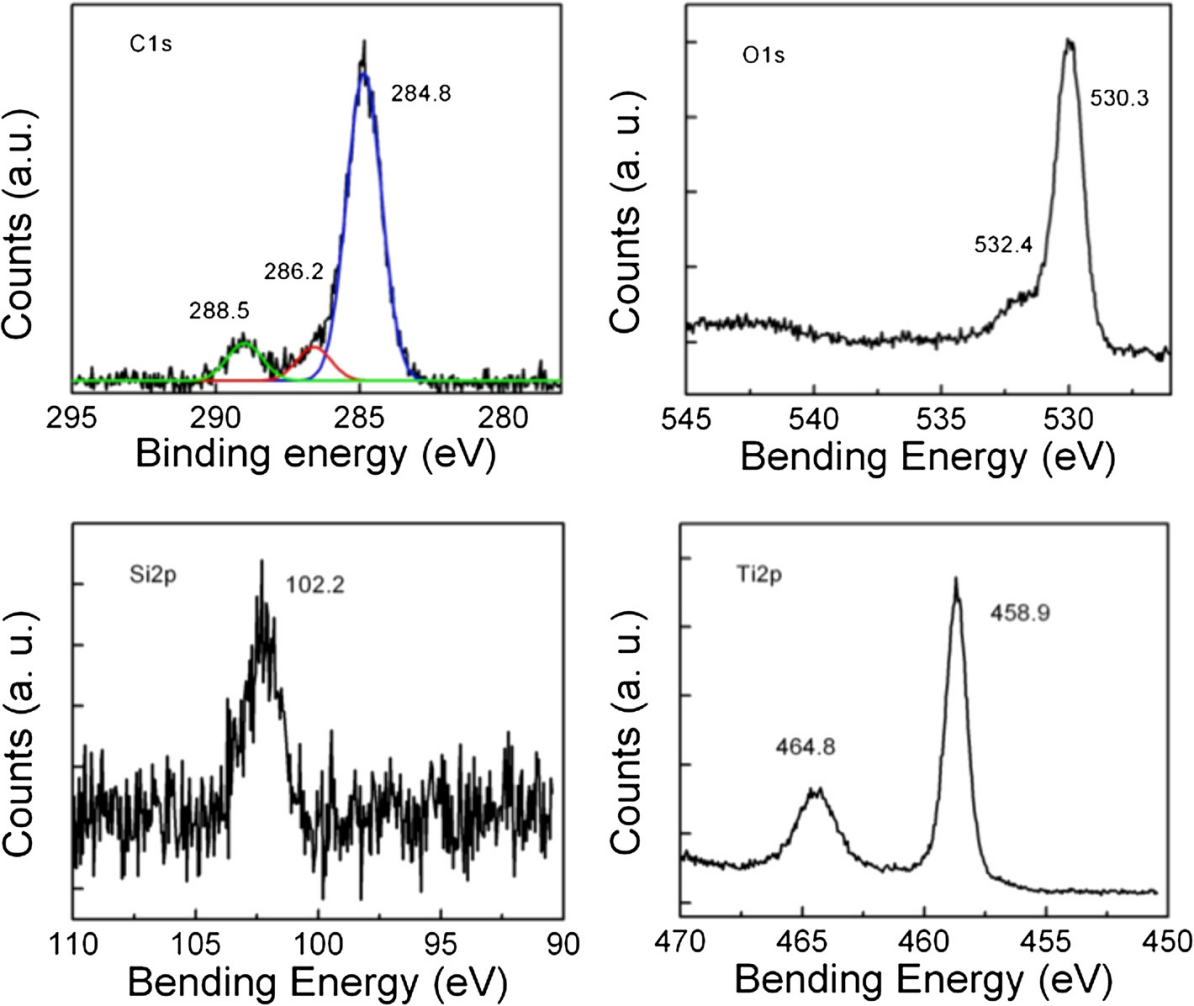
**C 1 s, O 1 s, Si 2p, and Ti 2p core levels of the XPS spectra obtained from the COOH-TiO**_**2**_**nanobelts.**

**Figure 5:**
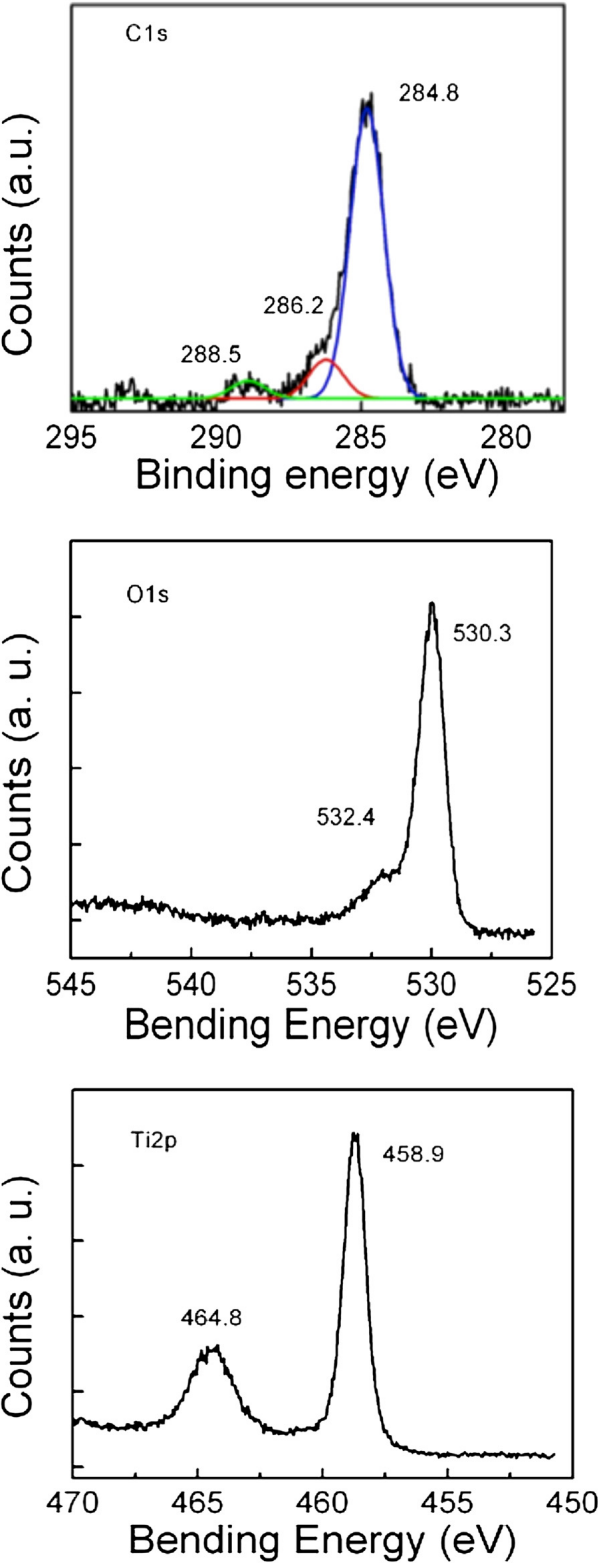
**Survey scan, C 1 s, O 1 s and Ti 2p core levels of the XPS spectra obtained from the humic acid-treated TiO**_**2**_**nanobelts.**

**Figure 6:**
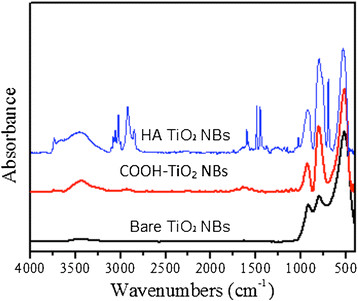
**FT-IR spectra of the bare, the COOH-terminated and the humic acid (HA)-treated TiO**_**2**_**nanobelts (NBs).**

Most of the TNB had lengths from 5-9 μm, and widths between 60–140 nm (Figure 1). After modification with carboxylic acid and humic acid, no evident change in the morphology was observed. The XRD pattern confirmed that the TNB had single anatase phase structure (Figure 2). XPS was used to analyze the surface chemistry of the nanobelts (Figures 3, 4, and 5). Figure 4 shows the XPS spectra of carboxylic acid-modified TNB. The doublet peaks at 464.8 eV and 458.9 eV confirmed that the core material (TiO_2_) was not altered [24]. The Si 2p at 102.2 eV was characteristic of silane. The C1s core level of XPS spectrum can be deconvoluted into three components that were assigned to C-C (284.8 eV), C-O (286.2 eV) and C = O (288.5 eV), respectively [7],[25], which indicated the successful functionalization of TNB with carboxylic acid. This was confirmed by the FT-IR band at 1710 cm^−1^ (C = O) (Figure 6). Humic acid (HA) is a mixture of various aromatic nuclei with phenolic and carboxylic substituents. Hence, the C 1 s and O 1 s XPS spectra of the HA-modified TNB in Figure 4 were similar to those of the carboxylic acid-functionalized TNB. The band between 3200 cm^−1^ and 3550 cm^−1^ were present in the FT-IR spectra of both the samples. The FT-IR band at 1740 cm^−1^ (C = O) also existed in the humic acid-treated sample. Unique to the HA-modified TNB, the FT-IR band at around 1500 cm^−1^ in Figure 6 was ascribed to the in-ring C–C stretch vibration of aromatic molecules; and the FT-IR band between 3100-3000 cm^−1^ corresponded to the C–H stretch of aromatic molecules. Therefore, the XPS and FT-IR spectra confirmed the presence of HA on the TNB surface. The zeta potential in the dispersion media was measured to be -13.2 mV, -12.6 mV and -12.1 mV for the bare, COOH- and HA-coated nanobelts, respectively (Table 1). In addition, the relative aggregate sizes (diameter ± range) of the TNB variants can be found in Table 1.

**Table 1:** Aggregate sizes and zeta potentials of all nanomaterials used in this study in the two applicable exposure mediums

	**RPMI culture media + 10%FBS**			**Dispersion media**		
***Particle***	***Average diameter (nm)***	***Range (nm)***	***Zeta potential (mV)***	***Average diameter (nm)***	***Range (nm)***	***Zeta potential (mV)***
TNB	386	128	−9.97	491	181	−13.2
COOH TNB	412	128	−10.93	404	124	−12.6
HA TNB	425	131	−10.87	363	118	−12.1
TNS	221	75	−9.89	205	86	−11.53
No particle	24*	19*	n/a	7*	1.4*	n/a

*measured in the noise of the signal.

### In vitro C57BL/6 mouse alveolar macrophage (AM) particle exposures

**Figure 7:**
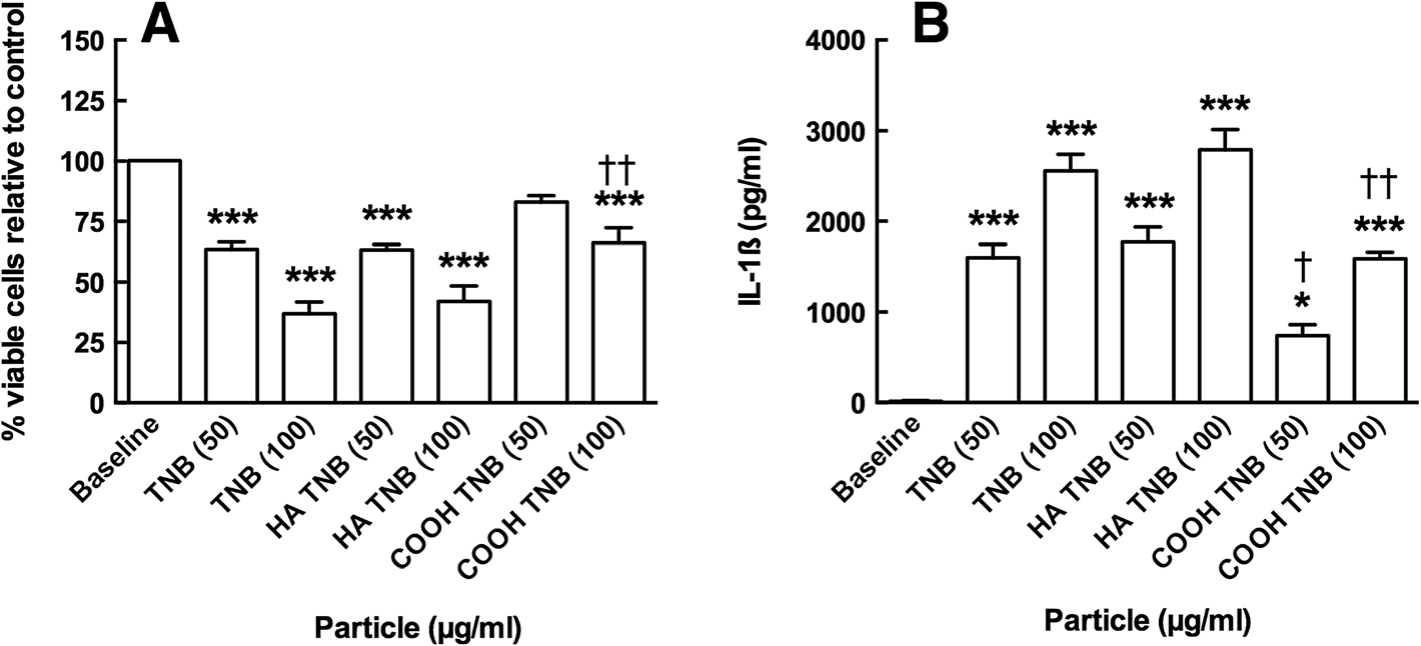
**Viability and IL-1β release following 24-h exposure to TiO**_**2**_**nanobelts in C57BL/6 alveolar macrophages co-cultured with 20 ng/mL LPS. A)** Mean ± *SEM* percent viable cells relative to no particle control. **B)** Mean ± *SEM* IL-1β release. Asterisks indicate significance at *** *P* < 0.001 or * *P* < 0.05 compared to baseline condition. Daggers indicate significance at †† *P* < 0.01 or † *P* < 0.05 compared to the two other nanobelt variants at the same concentration.

As described in Methods, isolated mouse alveolar macrophages (AM) were cultured for 24 hours with the TNB variants at two concentrations (50 and 100 μg/ml). Figure 7A shows the toxicity results. All of the TNB caused significant cell death at the highest concentration. However, TNB-COOH did not cause toxicity at the lower concentration and was significantly different than the other two TNB at both concentrations producing less cell death than TNB or TNB-HA. The IL-1β release results are shown in Figure 7B. Similar to the toxicity results, all of the TNB variants caused significant IL-1β release when co-cultured with LPS. This was indicative of NLRP3 inflammasome activation similar to the previous report with TNB [11]. Again, TNB-COOH deviated from the other two TNB by causing significantly less IL-1β release in exposed AM. Taken together, the results suggested that the TNB-COOH were significantly less bioactive than the other two TNB variants. The HA modification had no effect on TNB toxicity or NLRP3 inflammasome activation.

### TEM of TNB-exposed C57BL/6 AM

**Figure 8:**
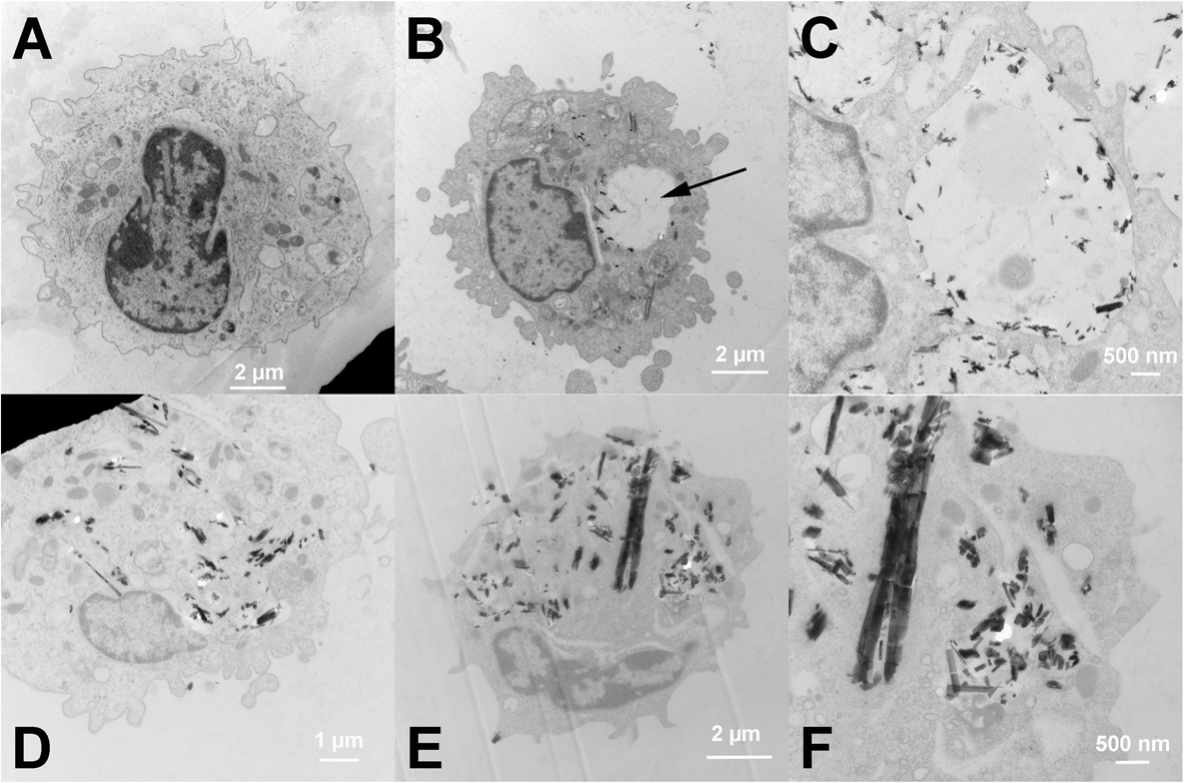
**TEM of TNP taken up by C57BL/6 alveolar macrophages 1.5 h in vitro post-exposure (25 μg/mL). A)** No particle control AM. **B)** TNB-exposed AM. Arrow indicates abnormal phagolysosomal enlargement. **C)** High magnification of enlarged phagolysosome in TNB-exposed AM. **D)** Humic acid-modified TNB-exposed AM. **E)** Carboxylated TNB-exposed AM. **F)** High magnification of carboxylated TNB-exposed AM. Black spotted/speckled areas indicate areas of particle retention in the cytoplasm of the macrophage cells.

Isolated AM from C57BL/6 mice were exposed to the various TNB for 1.5 hr in a suspension culture and processed for TEM imaging as described in Methods. Figure 8 shows the various treatments compared to the unexposed control AM in Figure 8A. TNB exposure resulted in organized particle uptake, with the resulting phagolysosome structure becoming unusually enlarged (Figure 8B). This was most likely due to the phagolysosomal rupture that precedes the NLRP3 inflammasome activation. Figure 8C is a close up of an affected phagolysosome area, and it was apparent that the TNB were in contact with the phagolysosmal membrane as opposed to the free open space within the lysosome, indicative of possible particle/membrane interactions. Figure 8D shows a TNB-HA-exposed AM with organized particle uptake, and without any enlarged lysosomal structures apparent. Figure 8E and F are TNB-COOH-exposed AM at low and high magnification respectively. Again, the phagolysosomal distortions were not obvious in these cells, but there was significant particle uptake mostly in organized interior structures.

### In vivo C57BL/6 short-term particle exposures

**Figure 9:**
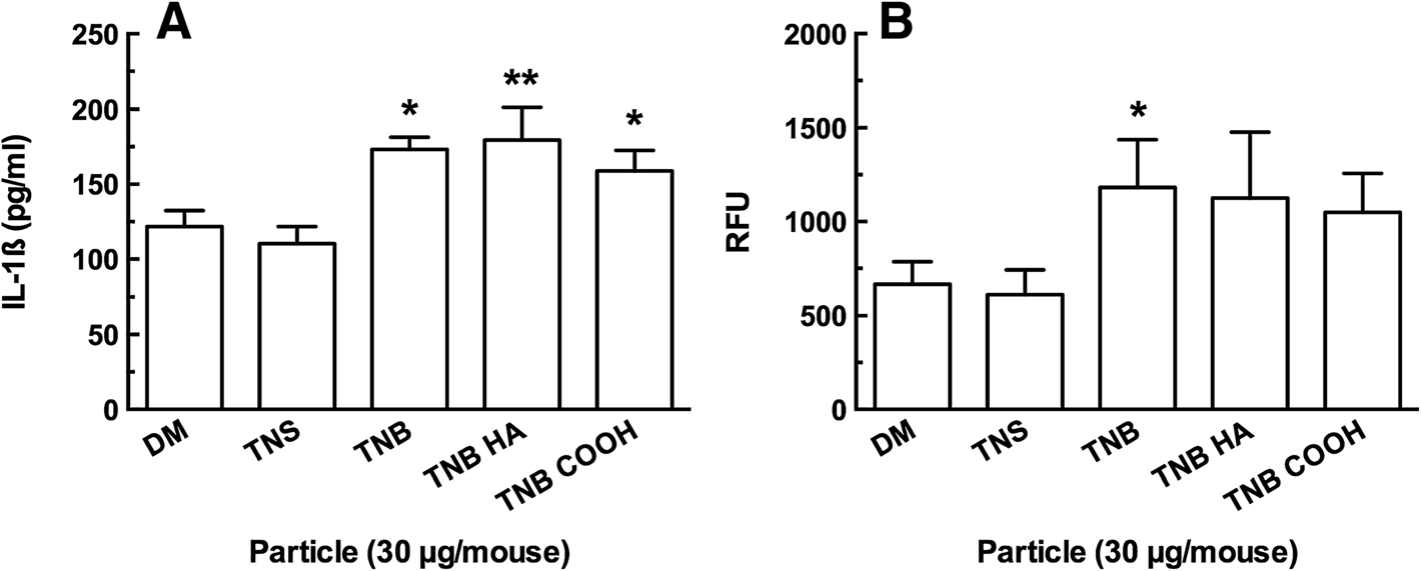
**Summary data of lavage fluid contents 4 h following TiO**_**2**_**nanobelt instillations in the lungs of C57BL/6 mice. A)** Mean ± *SEM* IL-1β release. **B)** Mean ± *SEM* relative fluorescence units (RFU) corresponding to active cathepsin B. Asterisks indicate significance at **P < 0.01, or *P < 0.05 compared to dispersion media vehicle (DM) and control particle TiO_2_ nanospheres (TNS).

Mice were instilled with one of the three TNB variants, dispersion media (DM), or a negative control particle TNS, for four (4) hr prior to lung removal and lavage. The isolated lavage fluid (first ml fraction) was assayed for IL-1β and active cathepsin B. Figure 9 shows the results of the *in vivo* exposures. Figure 9A shows that all three TNB caused significant IL-1β release at 4 hr compared to DM and TNS conditions. There was no difference between the three TNB, however. IL-1β was not elevated at 24 hrs (data not shown). Figure 9B shows the cathepsin B activity in isolated lavage fluid at 4 and 24 hr. While all three TNB variants showed increases in cathepsin B at both time points, only the TNB induced an elevation of cathepsin B that was significantly different than the DM condition. Taken together, the results presented in Figure 9 indicate that the acute inflammation caused by TNB exposure was mediated primarily by IL-1β release. The elevated cathepsin B levels in lavage fluid were probably due to TNB-damaged phagolysosomes and subsequent cell death.

### Comparing in vivo TNB exposures in C57BL/6 wild-type (WT) and IL-1R null mice

**Figure 10:**
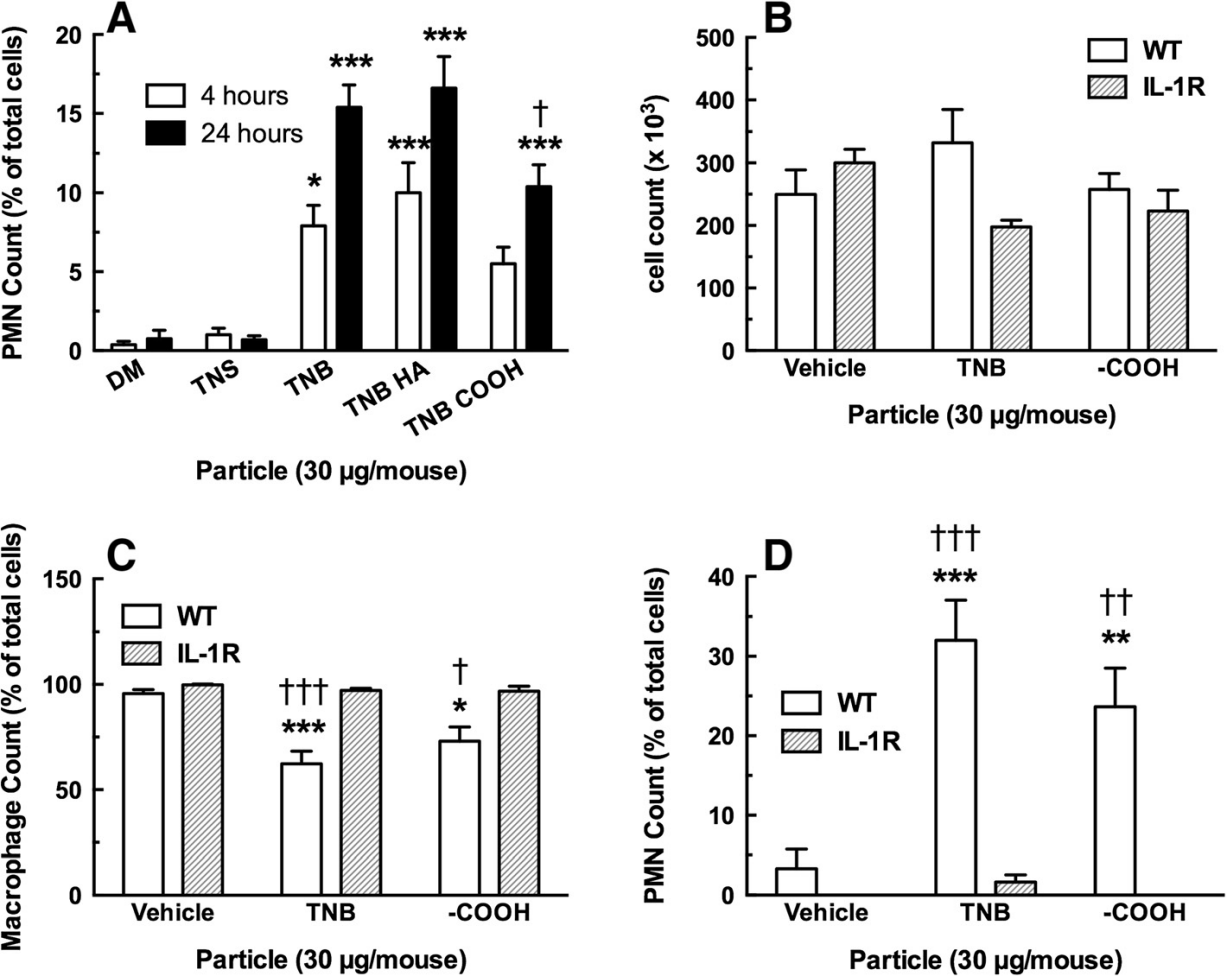
**Cell count and differentials 4 and 24 h following TiO**_**2**_**nanobelt instillations in C57BL/6 wild-type mice and IL-1R null mice (24 hr only). A)** Mean ± *SEM* neutrophil count at 4 and 24 h in WT mice only. **B)** Mean ± *SEM* total cell count at 24 h. **C)** Mean ± *SEM* alveolar macrophage count at 24 h. **D)** Mean ± *SEM* neutrophil count at 24 h. Asterisks indicate significance at ***P < 0.001, **P < 0.01 or *P < 0.05 compared to corresponding dispersion media vehicle (DM). Daggers indicate significance at ††† *P* < 0.001, †† *P* < 0.01, or † *P* < 0.05 compared to corresponding IL-1R particle exposure condition.

As stated above, mice were instilled with one of the three TNB variants, dispersion media (DM), or a negative control particle TNS, for 4 or 24 hr. All three of the TNB exposures produced significant elevations in neutrophils (PMN) at both 4 and 24 hr compared to both DM and TNS conditions (Figure 10A). Nevertheless, TNB-COOH caused significantly less PMN influx at 24 hr compared to TNB. This was consistent with the *in vitro* particle exposure results.

Since these initial *in vitro* and *in vivo* results indicated that the HA-modified TNB had no significant effects that differed from TNB, it was not used in the following experiments. Likewise, the TNS negative control particle was also not used. TNB or TNB-COOH were instilled in WT or IL-1R null mice for 24 hr, and lung lavage was conducted as described in Methods. The first set of experiments characterized the number and types of cells in the lung lavage fluid after 24 hr post-exposure. Figure 10B shows no significant deviations in the total cell counts following TNB instillations. Nevertheless, Figure 10C and D show expected decreases in AM and increases in PMN, respectively, only in the WT mice receiving TNB. The IL-1R null mice showed no acute inflammatory response. The absence of the IL-1 receptor had profound effects on the acute inflammation normally associated with titanium nanoparticle exposure. This was consistent with other results where IL-1β appeared to be the key inflammatory initiator associated with the original bioactive TNB [10],[11].

**Figure 11:**
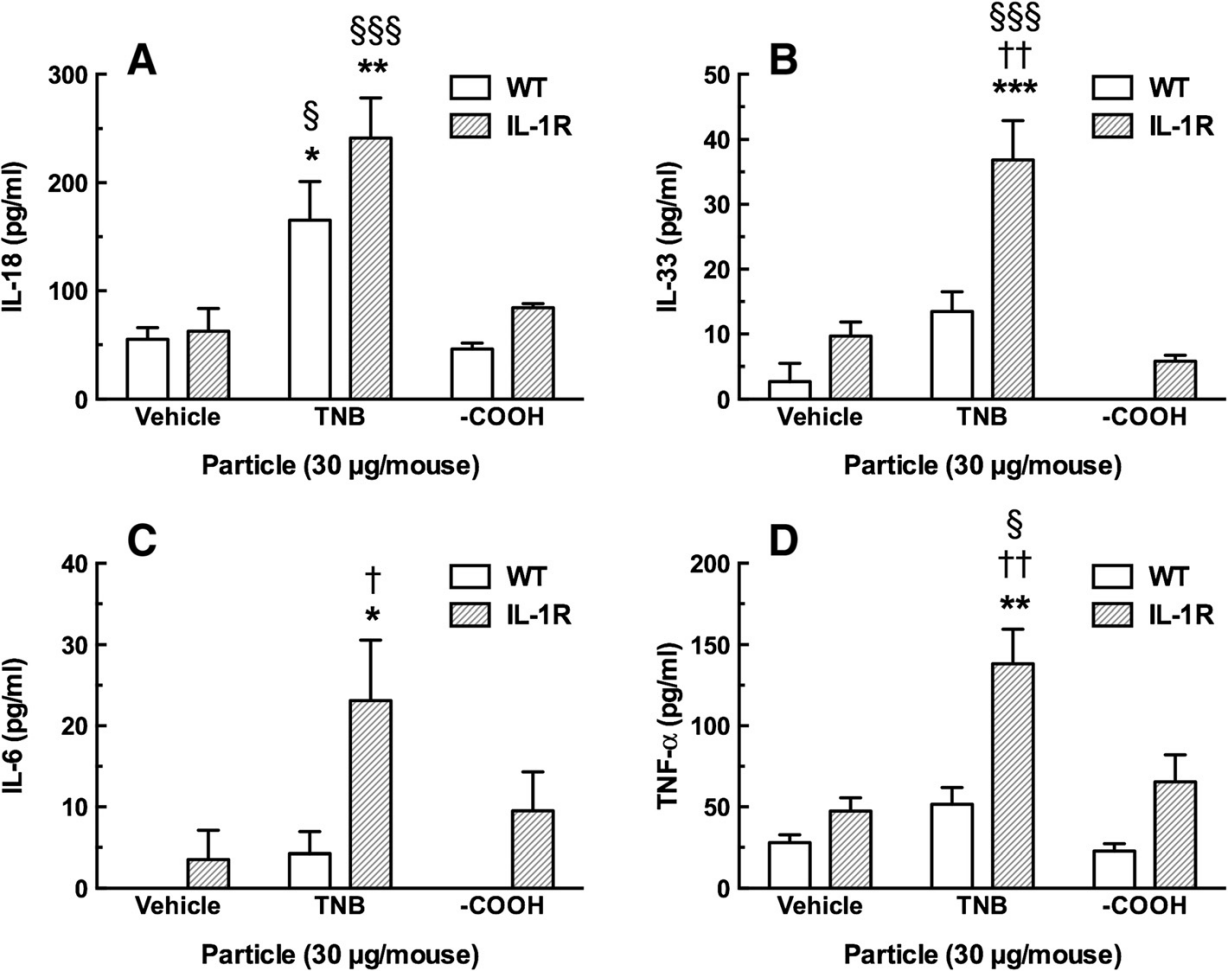
**Summary data of lavage fluid cytokines 24 h following TiO**_**2**_**nanobelt instillations in the lungs of C57BL/6 mice and IL-1R null mice. A)** Mean ± *SEM* IL-18 release. **B)** Mean ± *SEM* IL-33 release. **C)** Mean ± *SEM* IL-6 release. **D)** Mean ± *SEM* TNF-α release. Asterisks indicate significance at *** *P* < 0.001, ** *P* > 0.01 or * *P* < 0.05 compared to corresponding dispersion media vehicle (DM). Daggers indicate significance at †† *P* < 0.01, or † *P* < 0.05 compared to the corresponding wild-type particle instillation condition. Symbols §§§ indicate significance at *P* < 0.001, or § *P* < 0.05 compared to the corresponding TiO_2_ –COOH nanobelt (TNB COOH) instillation.

The 24-hr lung lavage fluid samples were also analyzed for cytokine content as shown in Figure 11. Significant IL-18 increase, seen in Figure 11A, occurred in both WT and IL-1R null mice treated with TNB indicating that activation of NLRP3 inflammasome occurred regardless of the presence or absence of IL-1R. In contrast, IL-33, IL-6 and TNF-α release was significantly higher in the TNB-exposed IL-1R lung lavage fluid samples as seen in Figure 11B, C and D, respectively, in IL-1R null mice than WT. These cytokine increases were significantly higher than the IL-1R DM control, the TNB WT exposure and the carboxylated TNB IL-1R exposure, indicating that the interaction of the particle type (TNB variants) and the strain (IL-1R) were critical for this effect. The cytokine results in the IL-1R null mice (elevated IL-6, IL-33 and TNF-α) might indicate an unknown alternative, compensatory mechanism initiating inflammation, since there was no IL-1 receptor to initially mediate an inflammatory response. The IL-1β release was at the limit of detection at 24 hr, and there were no significant differences with this cytokine at this time point (data not shown). The cytokine results, in general, were consistent with the observation that the original TNB were more bioactive than the modified TNB-COOH.

### Cytotoxicity and IL-1β release in the human THP-1 model

**Figure 12:**
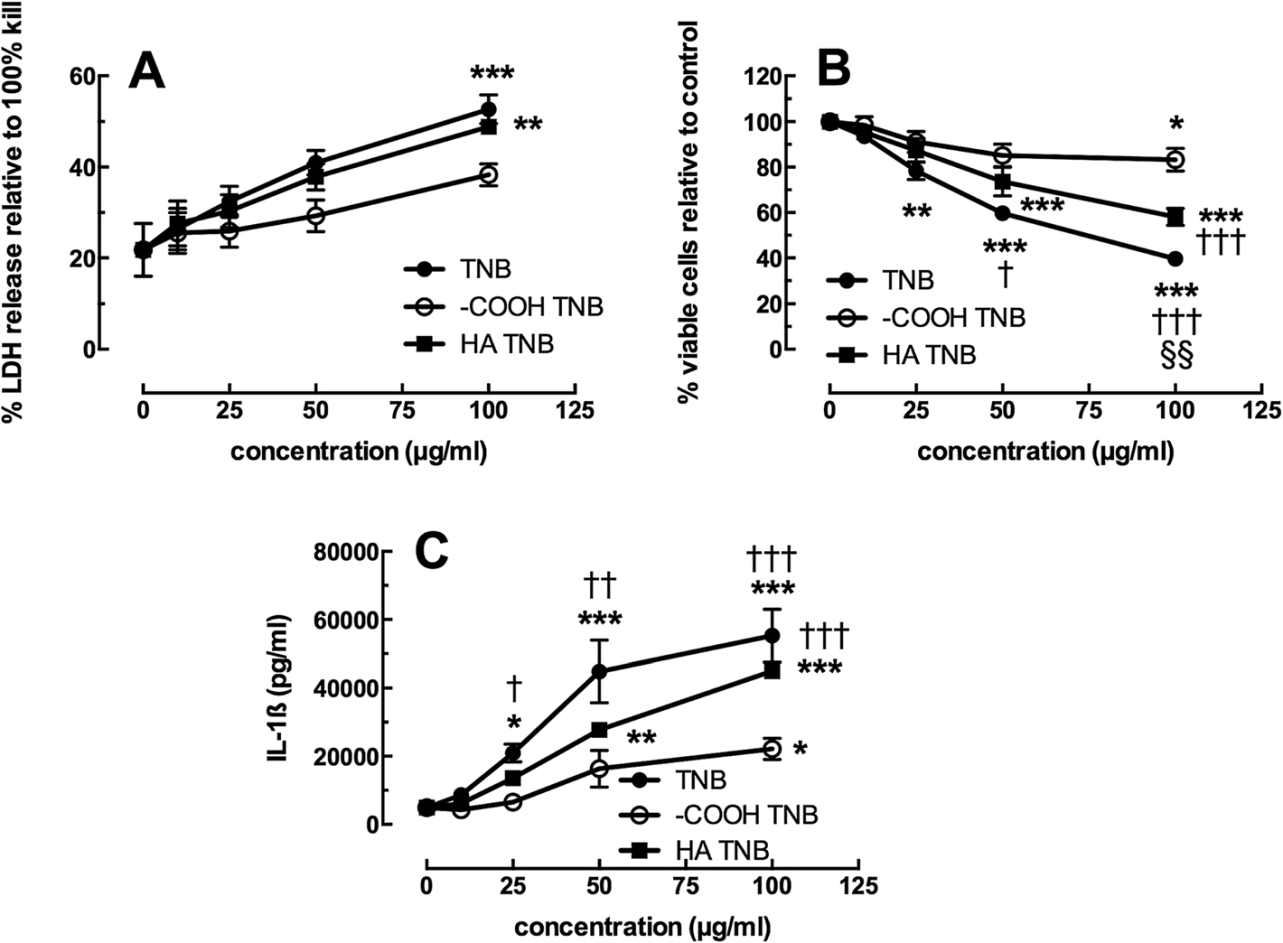
**Viability and IL-1β release following 24-h exposure to TiO**_**2**_**nanobelts in transformed human THP-1 cell line. A)** Mean ± *SEM* percent LDH release relative to total cell lysis. **B)** Mean ± *SEM* percent viable cells relative to no particle control. **C)** Mean ± *SEM* IL-1β release. Asterisks indicate significance at ***P < 0.001, **P < 0.01, or *P < 0.05 compared to no particle 0 μg/ml condition. Daggers indicate significance at ††† *P* < 0.001, †† *P* < 0.01 or † *P* < 0.05 compared to the TiO_2_ –COOH nanobelt (TNB COOH) at the same concentration. Symbols §§ indicate significance at *P* < 0.01 compared to the humic acid TiO_2_ nanobelt (TNB HA) condition at the same concentration.

The modified THP-1 model has previously been reported to be a good predictive model in the determination of nanoparticle bioactivity [21],[26] and it has been utilized by multiple laboratories for that purpose [27]. It was used here to confirm the above *in vitro* results with primary AM and help establish a high-throughput model system for future nanomaterial research. Figure 12A and B show the toxicity of the TNB variants in two different viability assays. The LDH assay in 12A shows a dose-dependent increase in LDH release for all three particles with TNB-COOH having the smallest effect. There was no difference between TNB and TNB-HA. Figure 12B using the MTS assay shows similar toxicity data, with the exception that TNB were slightly more toxic than TNB-HA. TNB-COOH was still the least toxic form consistent with all previous results. IL-1β release shown in Figure 11C was a dose-dependent increase for all three TNB variants with TNB being the most bioactive followed by TNB-HA and then by TNB-COOH. This data was also consistent with the *in vitro* data obtained in the mouse AM model. Taken together, it was apparent that TNB bioactivity in this model could be altered by surface modifications. In addition, it was apparent that COOH modification was effective at reducing the overall bioactivity in TNB exposures. Therefore, the THP-1 model was once again shown to be a good alternative to primary cell exposure models and also a good predictor of *in vivo* ENM-induced bioactivity.

### Inflammasome activity in other murine AM models

**Figure 13:**
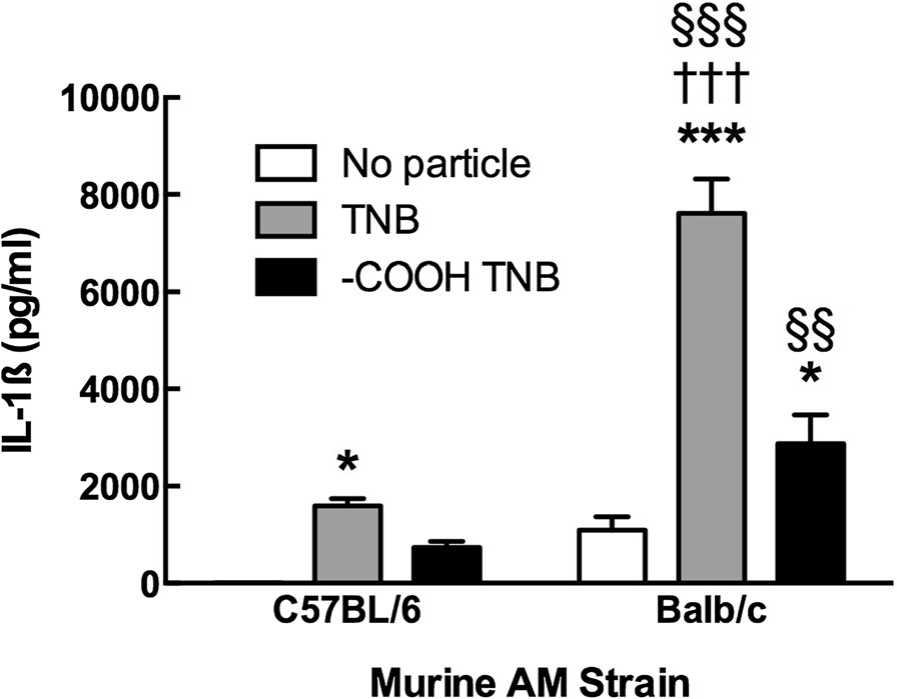
**IL-1β release following 24-h exposure to TiO**_**2**_**nanobelts in comparative murine AM cultures co-stimulated with 20 ng/mL LPS.** Data indicate mean ± *SEM* IL-1β release in isolated culture media. Asterisks indicate significance at ***P < 0.001, or *P < 0.05 compared to no particle 0 μg/ml condition. Daggers indicate significance at ††† *P* < 0.001 compared to the -COOH TiO_2_ nanobelt (-COOH TNB) exposure in the Balb/c AM. Symbols indicate significance at §§§ *P* < 0.001, or §§ *P* < 0.01 compared to the C57BL/6 AM TiO_2_ nanobelt (TNB) exposure condition.

Some effects seen with ENM can be strain-specific. Therefore, an *in vitro* particle exposure identical to the one presented earlier with the isolated C57BL/6 AM was conducted with AM isolated from the Balb/c mouse strain. The results of a 24 hr co-culture of TNB or TNB-COOH (both at 50 μg/ml) and LPS with AM are presented in Figure 13. The data are contrasted with the results from the C57BL/6 AM at the same particle concentration. BALB/c AM showed a qualitatively similar response to C57BL6 AM, as there was a greater IL-1β release in response to the TNB compared to TNB-COOH. Nevertheless, the BALB/c AM IL-1β release was 3 to 4 times greater than C57BL/6 AM IL-1β release. This indicated a significant strain difference in the response to TNB, but regardless of strain the effects are similar.

## Discussion

Nanotechnology is a rapidly developing field in the 21st century, and the commercial use of nanomaterials for novel applications is increasing exponentially [28]. Current expectations are that the field of nanotechnology has the capabilities to produce huge impacts on society. Therefore, it is important to identify any adverse effects of nanomaterials on human health and understand what modifications of nanomaterials will improve safety. The modifications can take one of many possible forms including shape, length, rigidity, hydrophobicity, and any number of various surface additions for specific purposes. We had previously reported that shape and length of TiO_2_ nanomaterials profoundly affected both cytotoxicity and ability to induce the release of inflammatory mediators *in vitro*[11] and cause inflammation *in vivo*. Furthermore, a number of groups have reported that carboxylation of carbon nanotubes decreased cytotoxicity and release of inflammatory mediators *in vitro* and decreased inflammation *in vivo*[18],[22],[27],[29],[30]. Therefore, it was important to determine if surface modification of highly bioactive TiO_2_ nanomaterials such as TNB could produce a similar trend in decreased bioactivity as occurred with carbon nanotubes.

This study utilized rigid TNB that were surface modified with -COOH groups or HA. Carboxylation was done to decrease bioactivity. HA modification was done to evaluate the effects of what might occur as part of the life cycle of TNB. HA is a main component of “humic substances”, which are major organic constituents of soil (humus), peat, coal, many upland streams, dystrophic lakes, and ocean water. Therefore, during the life cycle of TNB it is highly likely that they would be coated with HA that might result in a modification of bioactivity. The materials that were used in this study were fully characterized in order to confirm the surface modifications. The combination of XPS and FT-IR were important in characterizing all three forms of the anatase TNB. The XPS and FT-IR analysis confirmed the surface functionalization of TNB to TNB-COOH or TNB-HA.

Overall, the results demonstrated that carboxylation was effective in decreasing bioactivity of TNB both *in vitro* and *in vivo*. TNB-COOH was less toxic and less bioactive (Figure 7A and B, respectively) than either TNB or TNB-HA. Furthermore, there was a good correlation between the *in vitro* findings and the acute inflammatory response *in vivo*. TNB-HA were not distinguishable from TNB in either the *in vitro* or *in vivo* results with the exception of the TEM findings where TNB-HA appeared to behave more like TNB-COOH (Figure 8). The TEM results suggest that there were no qualitative differences in AM uptake of the three TNB variants. Therefore, any observed difference in particle toxicity and/or bioactivity was probably not due to a differential particle uptake in the AM, although this will need to be confirmed by a quantitative assessment at some point. TEM results also suggest that the TNB could be interacting with the phagolysosomal membranes, which would be consistent with the increased phagolysosomal disruption and release of cathepsin B (Figure 9B) into lung lavage fluid that was only significant for TNB.

Nanomaterials have been reported to modify rates of autophagy [13]. Although autophagy was not examined in this study, it could help explain the observations in the relative potency of the three NB. An increase in autophagy would result in increased degradation of the NLRP3 inflammasome and related components of the inflammatory pathway [13],[31],[32]. Therefore, it is possible that the increased activity of the bare NB could be a combination of lysosomal membrane permeability activating the NLRP3 inflammasome combined with a greater inhibition of autophagy than might occur with TNB-COOH.

The *in vivo* results were consistent with the *in vitro* activation of the NLRP3 inflammasome resulting in an acute inflammatory response. All three TNB variants released some IL-1β *in vivo* although the effect of COOH was the lowest. In addition, all three variants caused release of cathepsin B with only the effect of TNB being significant. All three TNB increased some PMN recruitment, however, the effect of TNB-COOH was not significant at 4 hr and while there was a significant increase in PMN recruitment at 24 hr by all three TNB, the TNB-COOH inflammatory response was the lowest. The difference in activity between TNB and TNB-COOH was again confirmed in studies comparing WT to IL-1R null mice (Figure 10), however, PMN recruitment was essentially eliminated in mice without IL-1R. This finding provides additional support for the critical role of IL-1β in mediating the acute inflammatory response and provides further rationale for examining the ability of nanomaterials to activate NLRP3 inflammasome *in vitro* as a screening tool to rank the biological activity of nanomaterials.

Extracellular release of IL-1β and IL-18 both require NLRP3 activation and Caspase-1 activation. The *in vivo* studies demonstrated very clear discrimination between the activity of TNB and TNB-COOH with IL-18 in lung lavage fluid (Figure 11A). Therefore, IL-18 might be a better tool to discriminate NLRP3 activation *in vivo* than IL-1β, most likely due to the short duration of IL-1β elevation *in vivo*. TNB also caused the release of TNF-α, IL-33 and IL-6 into the lung lavage fluid, while TNB-COOH had minimal to no effect. In contrast to the release of IL-18, the release of TNF-α, IL-33 and IL-6 were all profoundly enhanced in IL-1R mice compared to WT. This finding was unexpected, but suggests that IL-1R mice have compensatory mechanisms to release inflammatory cytokines in the absence of the ability for IL-1β signaling. Nevertheless, the importance of IL-1β to induce neutrophil recruitment was apparent from these studies.

Previous results have demonstrated that THP-1 cells provide good correlation with primary AM and could serve as a rapid screening tool for *in vivo* outcomes [18],[29]. The results in the current study confirm those findings since the differences in cytotoxicity and bioactivity among the three TNB were readily apparent using THP-1 cells. An important difference in the outcome between THP-1 and primary AM was that there was a reduction in the bioactivity due to HA, that was not apparent using primary AM. The difference might be due to the greater magnitude of the response using THP-1 cells, which allows more discrimination in the response. This suggests that the HA modification of TNB might be causing similar effects as surface carboxylation, but to a lesser extent. It was originally anticipated that HA would have a similar effect to carboxylation since it would add an increased negative surface charge to TNB surfaces. However, these anticipated effects were not apparent with primary AM or *in vivo*. Nevertheless, the subtle effect of HA was apparent using the more responsive THP-1 cells. These results also suggest that the presence of HA on nanomaterials could serve to make them less bioactive during their lifecycle.

Another option to test nanomaterials might be to utilize other murine strains as a more responsive model of primary AM. The results demonstrated that in fact primary AM from BALB/c mice were much more responsive to the TNB than AM from C57Bl/6 mice. The differences in responsiveness present an opportunity to examine critical points of regulation of the inflammatory responses and genetic variations. However, most of the various transgenic mice are on the C57Bl/6 background (e.g., IL-1R null), limiting the ability to explore mechanisms requiring transgenic models that are less common on BALB/c.

## Conclusions

Taken together, the results demonstrated that carboxylation of TNB significantly decreased their toxicity and ability to induce an inflammatory response. This was evident both *in vitro* and *in vivo*. Furthermore, the differences in activity were confirmed using THP-1 cells and AM from BALB/c mice. Overall, the relative activity of the three TNB appears to be TNB ≥ TNB-NB > > TNB-COOH. These findings are consistent with previous studies using carbon nanotubes and suggest that surface functionalization by carboxylation is an effective approach to decrease potential human health effects of exposure to nanomaterials. The findings are also consistent with previous work demonstrating the importance of the NLRP3 inflammasome in mediating the inflammatory response of ENM and the value of *in vitro* studies in serving as a mechanism-based screening tool to decrease the reliance on animal studies especially with the use of transformed THP-1 cells.

## Methods

### Preparation of materials

#### Chemicals

All TNP were purchased from Sigma-Aldrich (St Louis, MO). Humic acid (HA) was obtained from Alfa Aesar (Ward Hill, MA). Triethoxysilylpropyl succinic anhydride (TESPSA) was purchased from Gelest, Inc (Morrisville, PA). NaOH, HCl and 5% wt of tertramethylammonium hydroxide pentahydrate (TMAOH) were purchased from VWR (Randor, PA). All the chemicals were used without further modification.

#### Synthesis of bare TNB

First, 1.2 g of anatase TNP was added in 85 mL of 10 M NaOH aqueous solution with vigorously stirring for 1 h. Then the mixture was then transferred to a 100 mL flask and placed in a teflon-lined stainless steel autoclave and heated at 200°C for 24 h. After the hydrothermal processing, the products were washed by HCl and deionized (D.I.) water untill the pH reached ~7.0. The resulting H_2_Ti_3_O_7_ NB was dried in a vacuum oven at 80°C overnight, and then calcined in a quartz tube furnace at 700°C with a ramp rate of 1°C /min.

#### Synthesis of COOH-terminated TNB

COOH-functionalized TNB were prepared by surface modification of the bare TNB. In order to facilitate the adsorption of the hydroxyl group, 1.0 g of TNB was immersed into 10 mL of D.I. water, and the pH value was adjusted to 11 by adding 5% TMAOH resulting in the TNB terminated with the hydroxyl group. The product was then washed with methanol twice to remove the excessive TMAOH. The TMAOH-treated TNB were dried in a vacuum oven at RT. Afterwards, 3 mL of TESPSA were added to the TiO_2_ suspension in 20 mL of toluene. The mixture was further refluxed with an argon flow at 110°C for 24 h with vigorous stirring, and then washed three times by methanol to remove the free TESPSA. The resulting product was dried in a vacuum oven.

#### Humic acid-treated TNB

20 mg of TNB was mixed with a solution of 1 mL of aqueous solution containing 2.0 g/L HA and 19 mL of phosphate buffered saline (PBS) buffer solution (1 mM NaH_2_PO_4_ and 10 mM NaCl). The suspension was shaken for 48 h to reach the sorption equilibrium, and then centrifuged and dried in a vacuum oven at RT.

### Characterization of materials

The morphology of the TNB was observed with field-emission scanning electron microscope (SEM, JSM-7600 F, JEOL Ltd., Japan). The crystal structure of samples was identified by PANalytical X-ray diffractometer (XRD, PANanalytical, Westborough, MA). The chemical structure of surface-modified TiO_2_ was analyzed by X-ray photoelectron spectra (XPS)(PHI 5000 Versa Probe system Physical Electronics, Chanhassen, MN); and the deconvolution of XPS peak was carried out using the PHI Multipak™ software with the Gauss-Lorentz line and Shirley background subtraction (Physical Electronics, Chanhassen, MN). The monolayer on the TNP was further characterized by Fourier transform infrared spectra (FTIR), and measured under the transmission mode with a Thermo Nicolet 6700 spectrometer (ThermoFisher, Houston, TX). The zeta potential and aggregate size of the ENM was measured using electrophoretic light scattering with a Malvern Zetasizer Nano-ZS instrument (Malvern Instruments Ltd., Worcestershire, UK). The Malvern software established a number of pseudo-replicate runs (usually 10 to 20) to establish one value and these were repeated 3 times for an average value. The standard deviations of these data were always less than 10% of the means, and most were less than 5% indicating very little variance in the replications.

### In vitro experimental procedures

#### Animals

C57Bl/6, IL-1R null on C57BL/6 background, and BALB/c (2-months old, male) were housed in controlled environmental conditions (22 ± 2°C; 30-40% humidity, 12-h light: 12-h dark cycle) and provided food and water *ad libitum*. All procedures were performed under protocols approved by the IACUC of the University of Montana.

#### TNB suspensions

All TNB were weighed and suspended in freshly constituted 7.5% bovine serum albumin (BSA)/phosphate buffered saline (PBS) at 5 mg/mL. Just prior to use, low-speed magnetic stirring at RT for 1 h mixed the TNB suspensions. Sonication was not used due to potential damage to the TNB structure.

#### Alveolar macrophage isolation

Mice were euthanized by sodium pentobarbital (Euthasol™ Virbac Corp, Fort Worth, TX), and the lungs with the heart were removed. Lung lavage was performed using ice-cold PBS (pH 7.4). Lung lavage cells were isolated by centrifugation (400 × *g*, 5 min, 4°C) and cell counts obtained using a Coulter Z2 particle counter (Beckman Coulter, Miami, FL).

#### Cell culture

The alveolar macrophages (AM) cells were suspended in RPMI media supplemented with 10% fetal bovine serum, 0.05 mM 2-mercaptoethanol, sodium pyruvate, and supplemented with an antimycotic/antibiotic cocktail (Mediatech, Manassas, VA). Cells were suspended at 1 × 10^6^ cells per mL and then lipopolysaccharide (LPS, Sigma, St Louis, MO) at 20 ng/mL was added to stimulate pro-IL-1β formation. A 100 μl sample (100,000 cells) of cells were exposed to each TNB (ex: high dose 100 μg/mL equivalent to 10 μg/10^5^ cells equivalent to 31.25 μg/cm^2^ (10 μg on .32 cm^2^)) and experiments were conducted in 96-well plates for 24 h in 37e°C water-jacketed CO_2_ incubators (ThermoForma, Houston, TX). Particle concentrations ranged from 0, 10, 25, 50, 100 μg/mL. Media was collected for IL-1β assay and cell viability was determined by MTS assay.

#### Toxicity assay

Cell viability was determined by MTS reagent using the CellTiter^96^ assay (Promega, Madison, WI) according to the manufacturer’s protocol, with one exception described below. This assay used a colorimetric dye read by a colorimetric plate reader (Molecular Devices, Sunnyvale, CA). In order to avoid artifacts in the optical density values, steps were taken to remove the MTS reagent (transferring it into another plate) from the cell/particle mixture adhered to the plate bottom. The formation of bubbles was avoided and the plate was read at 490 nm.

### In vivo mouse 4 and 24 hr exposures

#### TNB suspension

Suspensions of TNB were prepared in dispersion medium (DM; Ca^2+^ and Mg^2+^-free phosphate buffered saline, pH 7.4, supplemented with 5.5 mM D-glucose, 0.6 mg/mL mouse serum albumin, and 0.01 mg/mL 1,2-dipalmitoyl-sn-glycero-3-phosphocholine) as previously described by our laboratory [33]. Suspensions of TNS were sonicated (5 W, 15 min) while TNB were mechanically stirred for 1 h.

#### Animals

Male C57BL/6 J mice (6 weeks old) were obtained from Jackson Laboratories (Bar Harbor, ME). Mice were housed one per cage in polycarbonate isolator ventilated cages, which were provided HEPA-filtered air, with fluorescent lighting from 0700 to 1900 h. Autoclaved Alpha-Dri virgin cellulose chips and hardwood Beta-chips were used as bedding. Mice were monitored to be free of endogenous viral pathogens, parasites, mycoplasms, Helicobacter and CAR Bacillus. Mice were maintained on Harlan Teklad Rodent Diet 7913 (Indianapolis, IN), and tap water was provided ad libitum. Animals were allowed to acclimate for at least 5 days before use. All animals used in this study were housed at the National Institute for Occupational Safety and Health (Morgantown, WV), which is an AAALAC-accredited, specific pathogen-free, environmentally controlled facility. All procedures involving animals were approved by the NIOSH Institutional Animal Care and Use Committee.

#### Pharyngeal aspiration exposure of mice

Suspensions of TNP were prepared in DM as described above. Mice were anesthetized with isoflurane (Abbott Laboratories, North Chicago, IL), and, when fully anesthetized, the mouse was positioned with its back against a slant board and suspended by the incisor teeth using a rubber band. The mouth was opened, and the tongue gently pulled aside from the oral cavity. A 50 μL aliquot of particle suspension was pipetted at the base of the tongue, and the tongue was restrained until at least 2 deep breaths were completed (but for not longer than 15 sec). Following release of the tongue, the mouse was gently lifted off the board, placed on its left side, and monitored for recovery from anesthesia. Mice received a single dose of either DM (vehicle control), or 30 μg/mouse of TNS, TNB, TNB-COOH or TNB-HA.

#### Whole lung lavage

At 4 and 24 h post-exposure, mice were euthanized with an i.p. injection of sodium pentobarbital (>100 mg/kg body weight) followed by exsanguination. A tracheal cannula was inserted and whole lung lavage (WLL) was performed through the cannula using ice cold Ca^2+^ and Mg^2+^-free phosphate buffered saline, pH 7.4, supplemented with 5.5 mM D-glucose (PBS). The first lavage (0.6 mL) was kept separate from the rest of the lavage fluid. Subsequent lavages, each with 1 mL of PBS, were performed until a total of 4 mL of lavage fluid was collected. WLL cells were isolated by centrifugation (650 × *g*, 5 minutes, 4°C). An aliquot of the acellular supernatant from the first WLL (WLL fluid) was decanted and transferred to tubes for analysis of lactate dehydrogenase (LDH) and albumin. The acellular supernatants from the remaining lavage samples were decanted and discarded. WLL cells isolated from the first and subsequent lavages for the same mouse were pooled after resuspension in PBS, centrifuged a second time (650 × *g*, 5 min, 4°C), and the supernatant decanted and discarded. The WLL cell pellet was then resuspended in PBS and placed on ice. Total WLL cell counts were obtained using a Coulter Multisizer 3 (Coulter Electronics, Hialeah, FL), and cytospin preparations of the WLL cells were made using a cytocentrifuge (Shandon Elliot Cytocentrifuge, London). The cytospin preparations were stained with modified Wright-Giemsa stain, and cell differentials were determined by light microscopy.

#### WLL fluid cathepsin activity

As previously described by our laboratory [23], to determine total and B-specific cathepsin activities the following assay components were mixed in a 96-well plate using PBS as diluent: first WLL fluid (50 μL), 2 μg Z-LR-AMC (fluorogenic Peptide Substrate, R & D systems, Minneapolis, MN, USA) ± 66 μM inhibitor (Z-Phe-Phe-FMK, MBL International, Woburn, MA, USA) in a total volume of 150 μL. The assays samples were incubated at 37°C for 1 h then fluorescence was measured using a plate reader at 380 nm excitation and 460 nm emission. Cathepsin-B specific activity was calculated as follows: relative fluorescence units (RFU) from assay without inhibitor minus the assay with inhibitor.

### In vivo mouse exposures for IL-1R experiments

All TNB were suspended in DM as described above. Mice were exposed to TNB by oro-pharyngeal aspiration. Briefly, the mice were anesthetized using inhalation isoflurane and a volume of 25 μL of particle suspension (1.2 mg/Kg or 30 μg/ 25 g mouse) was delivered into the back of the throat. By holding the tongue to the side, the solution was aspirated into the lungs. After 24 h the lungs were removed from exposed mice and WLL was performed with cold PBS as described above. The first mL of lavage fluid was centrifuged (1 min at 7000 rpm in microfuge), and isolated for assay of IL-1β, IL-6, IL-18, IL-33 and TNF-α. The isolated cells from these samples, and the remaining lavage sample, were cultured with and without LPS (20 ng/mL) for an additional 24 h in a manor described above. The media was isolated and assayed for IL-1β, IL-6, IL-18, IL-33 and TNF-α. Cell differentials were determined by centrifuging a small sample (35 × 10^3^ cells) on to positively charged glass slides in a cytocentrifuge at 400 x *g* for 5 min (Shandon Cytospin 3, Thermo Fisher, Houston, TX). The slides were then stained in a Hematek slide stainer (Bayer Diagnostics, Dublin, Ireland) with a modified Wright-Giemsa stain (Protocol, Fisher, Houston, TX). The slides were allowed to dry. Differentials were conducted on a Zeiss microscope at 400x and 200 cell counts per slide.

### Electron microscopy

Isolated AM from C57BL/6 mice were exposed to TNP at 25 μg/mL for 1.5 h in suspension culture using 1.5 mL polypropylene tubes on a slowly rotating mixer (LabQuake Shaker, Lab Industries, Berkley, CA). The cells were washed once in PBS and resulting macrophage suspensions were fixed in 2.5% EM grade glutaraldehyde in cacodylate buffer at pH 7.2 (EMS, Electron Microscopy Sciences, Hatfield, PA). The cells were then rinsed in dH_2_O and resuspended in 1% osmium tetroxide (EMS) for 1 h and rinsed in dH_2_O. The cells were dried in a graded ethanol series followed by embedding of the cell pellet in epoxy resin. Thin sections were stained with 2% uranyl acetate (EMS) for 30 min at room temperature, rinsed in dH_2_O, and stained for 5 min with Reynolds lead citrate stain (EMS). The cells were imaged in a Hitachi H-7100 transmission electron microscope (Chula Vista, CA) at 75 kV.

### Cytokine assays

Mouse and human IL-1β DuoSets were obtained from R&D Systems (Minneapolis, MN) and ELISA assays performed according to the manufacturer’s protocol. IL-6, IL-33 and TNF-α DuoSet ELISA’s, and IL-18 capture and detection antibodies were also obtained from R & D Systems. The IL-18 ELISA, although developed in-house, was run similar to R & D Systems IL-33 DuoSet ELISA with regard for timings, diluents, standard curves, and washes. Lavage fluid samples were assayed without dilution. All plates were read at 450 nm and data expressed as pg/ml.

### Human THP-1 cell line culturing

THP-1 cells, a human monocytic cell line obtained from ATCC, were suspended in RPMI media (MediaTech, Manassas, VA) supplemented with 10% fetal bovine serum, 50 μM beta-mercapto ethanol, 1 mM sodium pyruvate, 250 ng/ml amphotericin B, and 100 U/ml penicillin and streptomycin (all supplements Media Tech, Manassas, VA), and cultured in 75 cm^2^ flasks at 37°C. The cells in suspension were differentiated into a macrophage-like cell by adding 150 nM Vitamin D_3_ (1α, 25-dihydroxy, EMD Millipore, Darmstadt, Germany) for 24 hr. The semi-adherent cells were scrapped with a rubber policeman in the existing media (Corning, Corning, NY). The cells were then centrifuged at 400 x *g* for 5 min, the resulting cell pellet was re-suspended in 1 mL of complete media, and a 40 μL sample was then counted on a Z2 Coulter Counter (Beckman Coulter, Miami, FL). The cells were suspended at 1 × 10^6^ cells/mL and a small amount of phorbol 12-myristate 13-acetate (5 nM PMA, sigma) and lipopolysacharride (10 ng/mL LPS, Sigma, St Louis, MO) was added. The PMA co-stimulation was necessary to stimulate aggressive phagocytosis of the TNB. The LPS co-stimulation was necessary to induce NF-αB translocation leading to pro-IL-1β synthesis for the NLRP3 inflammasome to cleave for IL-1β release in the transformed THP-1 model [12],[15]. Cells, at a volume of 350 μL, were then pipetted in to 1.5 mL microfuge tubes. The TNB conditions were added from 5 mg/mL concentrated stock suspensions to the cells. The TNB variants used a range of concentrations (0, 10, 25, 50 and 100 μg/ml). The resulting cell/particle suspension was mixed by pipette action. The cells were then transferred to 96-well tissue culture plates at 100 μL per well in triplicate (100 × 10^3^ cells/well), and cultured for an additional 24 h. All cultures were maintained in 37°C water-jacketed CO_2_ incubators (ThermoForma, Houston, TX). Viability and IL-1β levels were determined as described above with the exception of an additional viability assay for LDH (Promega, Madison, WI), which was run according to the manufacturer’s protocol.

### Statistical analyses

Statistical analyses involved comparison of means using a one or two-way *ANOVA* followed by Dunnett’s test or Bonferroni’s test to compensate for increased type I error. All probabilities were two-tailed unless otherwise stated. Statistical power was greater than 0.8. Statistical significance was defined as a probability of type I error occurring at less than 5% (*P* < 0.05). The minimum number of experimental replications was 3 – 8 depending on the experiment. Graphics and analyses were performed on PRISM 6.0.

## Competing interests

The authors have no competing interests to declare.

## Authors’ contributions

NW, CX, ML and FY were responsible for the preparation and characterization of the TNB. AH and DP were responsible for the experimental design. RH conducted the *in vitro* and some of the *in vivo* studies and drafted the manuscript with AH. DP and MW conducted some of the *in vivo* studies. All authors reviewed and approved of the manuscript.
